# Allele Specific Expression of the Transthyretin Gene in Swedish Patients with Hereditary Transthyretin Amyloidosis (ATTR V30M) Is Similar between the Two Alleles

**DOI:** 10.1371/journal.pone.0049981

**Published:** 2012-11-19

**Authors:** Nina Norgren, Urban Hellman, Bo Göran Ericzon, Malin Olsson, Ole B. Suhr

**Affiliations:** 1 Department of Public Health and Clinical Medicine, Umeå University, Umeå, Sweden; 2 Division of Transplantation Surgery, Karolinska Institutet, Stockholm, Sweden; Oslo University Hospital, Norway

## Abstract

**Background:**

Hereditary transthyretin (TTR) amyloidosis (ATTR) is an autosomal dominant disease characterized by extracellular deposits of amyloid fibrils composed of misfolded TTR. The differences in penetrance and age at onset are vast, both between and within populations, with a generally late onset for Swedish carriers. In a recent study the entire *TTR* gene including the 3′ UTR in Swedish, French and Japanese ATTR patients was sequenced. The study disclosed a SNP in the V30M TTR 3′ UTR of the Swedish ATTR population that was not present in either the French or the Japanese populations (rs62093482-C>T). This SNP could create a new binding site for miRNA, which would increase degradation of the mutated TTR’s mRNA thus decrease variant TTR formation and thereby delay the onset of the disease. The aim of the present study was to disclose differences in allele specific *TTR* expression among Swedish V30M patients, and to see if selected miRNA had any effect upon the expression.

**Methodology/Principal Findings:**

Allele-specific expression was measured on nine liver biopsies from Swedish ATTR patients using SNaPshot Multiplex assay. Luciferase activity was measured on cell lines transfected with constructs containing the *TTR* 3′ UTR. Allele-specific expression measured on liver biopsies from Swedish ATTR patients showed no difference in expression between the two alleles. Neither was there any difference in expression between cell lines co-transfected with two constructs with or without the TTR 3′ UTR SNP regardless of added miRNA.

**Conclusions/Significance:**

The SNP found in the 3′ UTR of the *TTR* gene has no effect on degrading the variant allele’s expression and thus has no impact on the diminished penetrance of the trait in the Swedish population. However, the 3′ UTR SNP is unique for patients descending from the Swedish founder, and this SNP could be utilized to identify ATTR patients of Swedish descent.

## Introduction

Hereditary transthyretin (TTR) amyloidosis (ATTR) is an autosomal dominant disease, where mutations in the *TTR* gene cause the normally stable tetrameric structure to dissociate into amyloigenic monomers that aggregate into fibrils in peripheral tissues. [1] Clinical symptoms often consist of a peripheral sensory-motor neuropathy and/or hypertrophic cardiomyopathy. [2] The phenotypic variations between different mutations and individuals, as well as different populations, are vast. In Sweden, the disease onset for carriers of the *TTR* V30M mutation is on average 56 years compared to the Portuguese V30M population’s average age at onset of 34 years. In addition, 5% of the Swedish and French V30M carriers will have developed symptomatic disease at the age of 40 compared with 56% for the Portuguese population. The explanation for these remarkable differences in the phenotypes has not been elucidated. [3], [4].

Since TTR is mainly synthesized in the liver, the main therapeutic modality is liver transplantation which removes the source of the mutated protein. However, new drugs against ATTR are under exploration in clinical trials, of which the TTR-stabilizing small molecule Tafamidis appears to delay disease progression. [5] Therapy focusing on siRNA, and antisense designed to bind to the *TTR* gene, and thereby reduce expression of the protein is also under development. [6], [7].

miRNA is a group of siRNA that consists of short endogenous RNA that regulates the expression of mRNA either by binding to the 3′ UTR of a transcript, thereby marking it for degradation by the RNA-induced silencing complex (RISC), or by binding to the mRNA, preventing translation of the protein. [8] Several studies have investigated the relationship between SNPs in the 3′ UTR of specific disease genes and differences in phenotype.[9]–[12] These studies disclosed that different SNPs in the 3' UTR of genes are associated with different phenotypes sometimes causing allele-specific expression resulting in differences in phenotypes.

**Table 1:** Clinical data on patients used in the study.

	Patients *n = 9*
Gender (male/female)	7/2
Age at transplantation (liver biopsy) (mean, range)	56 (35–69)
Age at diagnosis (<50 years/>50 years)	2/7
Duration of symptomatic disease before transplantation, years (mean/range)	4 (2–10)

In a previous study we sequenced the entire *TTR* gene including the 3′ UTR in Swedish, French and Japanese ATTR V30M patients. [13] The result of that study revealed a SNP (rs62093482C>T) in the V30M *TTR* 3′ UTR of the Swedish ATTR population that could not be found in the French or Japanese ATTR populations. The rs62093482-T SNP is also very uncommon in the Portuguese ATTR population [14] and in the normal population it is only found in 2% of the European population according to the 1000 Genomes database. We hypothesized that this SNP could be one of the explanations for the reduced penetrance in the Swedish population, through the actions of miRNA degrading the mutated TTR allele transcript thereby reducing tetrameric instability and reduce precursor protein availability for amyloid formation. Using bioinformatic methods we estimated four miRNA candidates that most likely bind to the mutated *TTR* allele although not the wild type *TTR*.

**Figure 1:**
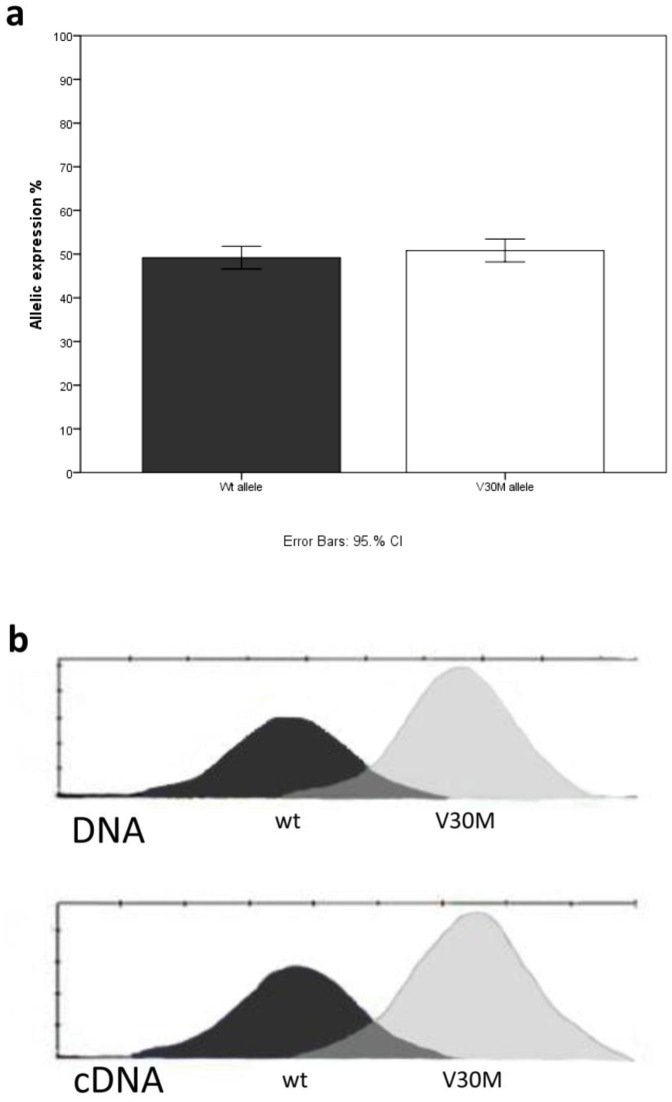
Allele specific expression from eight liver biopsies. **A**. mRNA expression from the V30M and wild type allele were measured using a SNapShot assay. All eight samples were measured three times in independent assays. For each sample, the peak height from the cDNA sample was normalized by the peak height from the DNA samples. The results are presented as the allelic ratio between wild type and V30M expression for each sample, with the total expression from both the wild type allele and V30M allele set to 100%. Data are shown as mean ±CI. No difference between the two alleles was noted. b. Representative example of DNA and cDNA samples after SNaPshot assay measurements.

The aim of the present study was to investigate whether any of the four miRNA from our previous study, or a liver endogenous miRNA, could reduce expression of the mutated *TTR* allele. We also wanted to investigate the allele specific expression in liver biopsies from nine transplanted Swedish ATTR patients, all expectedly carrying the T-SNP in the 3′ UTR.

**Figure 2:**
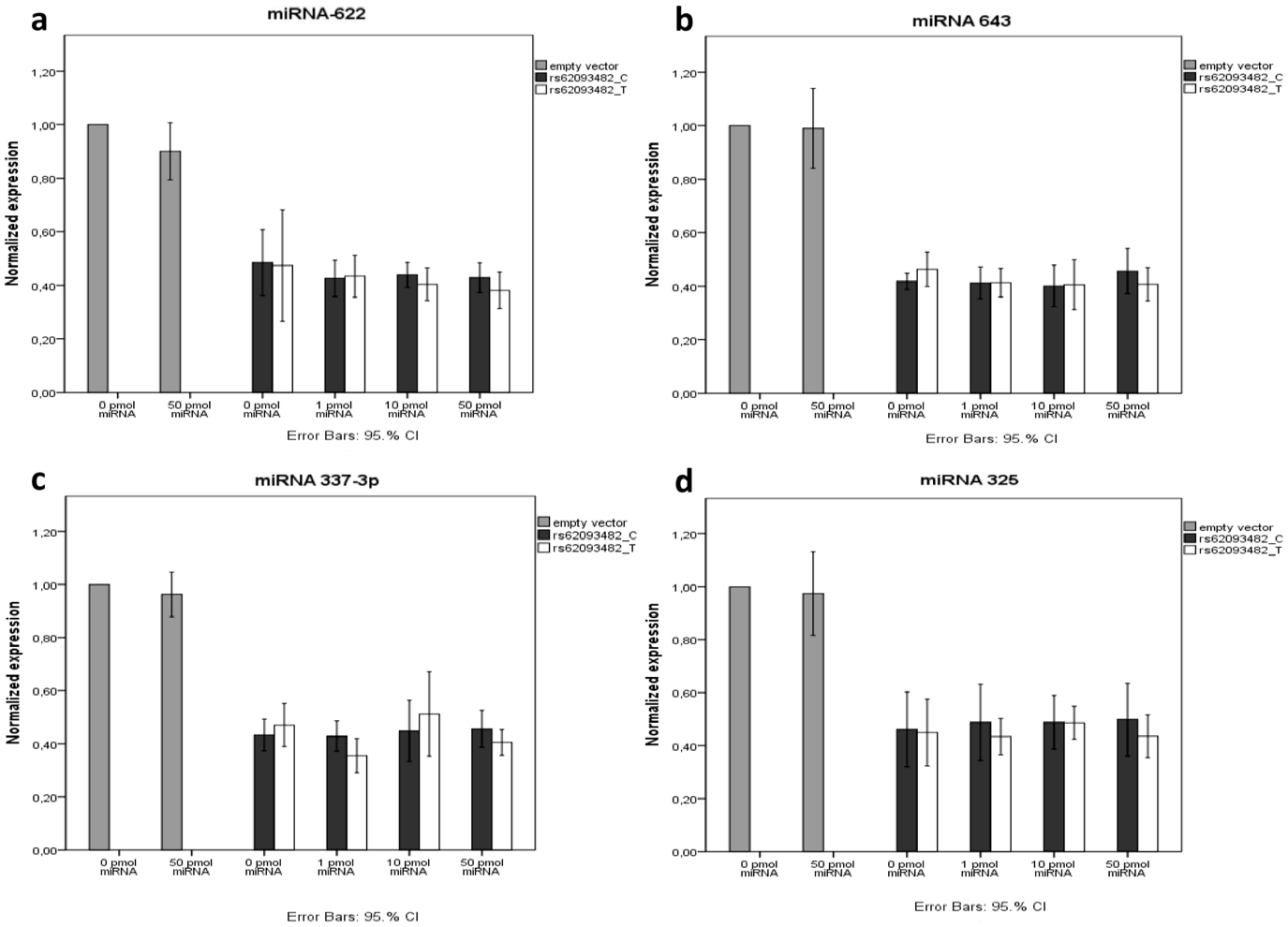
Luciferase activity in a COS-7 cell line co-transfected with the constructs (pmirGLO-TTR-3′UTR–C/T for rs62093482) together with different concentrations of miRNA. Luciferase activity was measured after 26 h, and normalized to a transfected empty pmirGLO vector. All experiments were made in triplicate, and data are shown as mean ±CI. a: miRNA-622 b: miRNA-643 c: miRNA-337-3p d: miRNA-325. No significant difference between the two constructs was seen regardless of concentration.

## Methods

### Material

Liver biopsies from nine heterozygous Swedish ATTR patients were taken during transplantation, and the biopsies were immediately transferred to RNAlater (Qiagen, Inc., Valencia, CA, USA), and thereafter stored in −80°C until analysis. Clinical data on patients can be found in Table 1.

**Figure 3:**
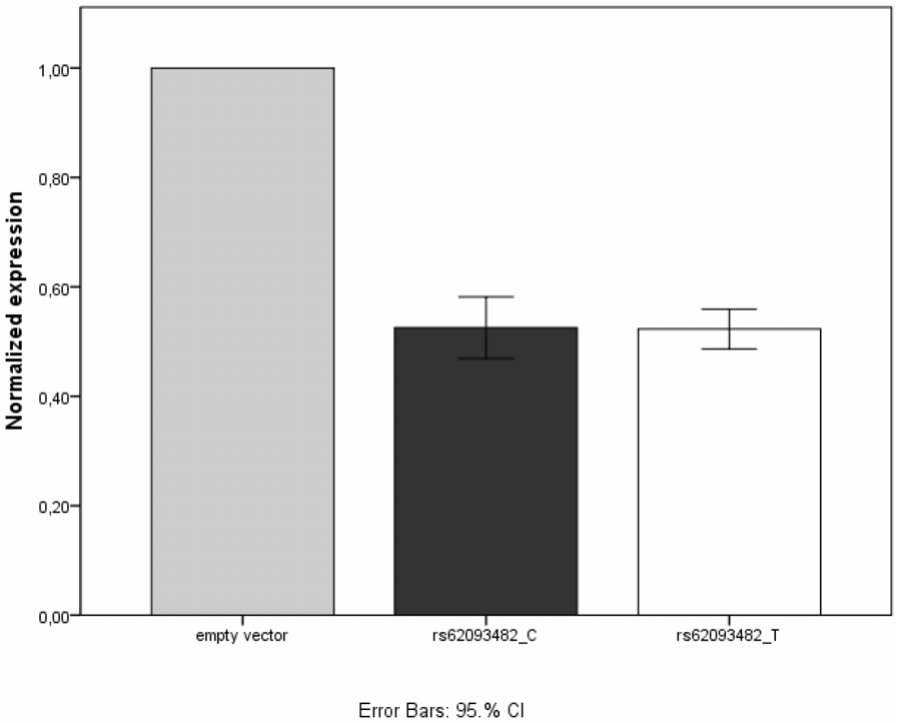
Luciferase activity in a HepG2 cell line transfected with either of the constructs (pmirGLO-TTR-3′UTR–C/T for rs62093482). Luciferase activity was measured after 40 h, and normalized to a transfected empty vector. Data are presented as the mean of 20 measures ±CI. No difference between the two constructs was seen.

### Cell Culture

COS-7 cells, an African green monkey kidney fibroblast-like cell line and HepG2 cells, a human cell line derived from liver tissue with hepatocellular carcinoma, were obtained from ATCC-LGC. The cells were cultured in Dulbecco’s modified Eagle’s medium containing 10% heat inactivated fetal calf serum and penicillin-streptomycin and contained at 37°C in 5% CO_2_.

### Allele Specific Expression

Total RNA was extracted from the liver biopsies using miRNeasy Mini Kit (Qiagen) according to the manufacturer’s protocol. DNA was simultaneously extracted using proteinase K and lysis buffer. cDNA was synthesized using Superscript II Reversed Transcriptase (Invitrogen) according to the manufacturer’s protocol. All samples were sequenced to confirm the V30M mutation and the SNP rs62093482-T. Primers were designed to amplify exon 2 of the TTR gene for the DNA samples (F: 5′CGGTGAATCCAAGTGTCCTC3′, R: 5′AGGTGTCATCAGCAGCCTTT3′), and for exon 2 and part of exon 3 for the cDNA samples to verify that no genomic DNA was interfering (F: 5′CGGTGAATCCAAGTGTCCTC3′, R: 5′CCATGCAGCTCTCCAGACTC3′). A SNaPshot Multiplex assay (Applied Biosystems) with the extension primer 5′TCCTGCCATCAATGTGGCC3′ was used according to the manufacturer’s protocol. Samples were run on a 3730xl DNA analyzer (Applied Biosystems) and the data were analyzed with GeneMapper v3.5 (Applied Biosystems). All samples were analyzed in three independent runs.

Peak height for both genomic DNA and cDNA were measured for each allele. The relative peak heights for the two alleles in genomic DNA were set to 1, and the relative peak height for cDNA was used for determination of allele expression (Figure 1b).

### pmirGLO-TTR-3′UTR Construction

Constructs were created by fusing the TTR 3′ UTR with the firefly luciferase gene in a pmirGLO vector (Promega) designed for miRNA analysis. The TTR 3′ UTR was first amplified from DNA from both a control person with the rs62093482-C-SNP and a patient homozygous for the rs62093482-T-SNP using standard PCR reactions with specific primers: (F:5′GAGCTAGCGTTCTCCTCCAGTGGACCTG3′, R:5′AGCAGCTGTGCCCACAGTAAAGAAGTGG3′). Primers used in the reactions were designed to incorporate restriction sites for Nhe1 and Sal1. The resulting fragments were purified from gel, and inserted into a pGMTeasy vector (Promega). These constructs were then digested with Nhe1 and Sal1, and finally inserted into the pmirGLO vector in the 3′ UTR of the firefly luciferase gene, creating the constructs pmirGLO-TTR-3′UTR-C and pmirGLO-TTR-3′UTR-T. All constructs were verified by direct sequencing.

### Luciferase Assay

Luciferase activity was measured in the cell line COS-7. The cells were seeded at 5×10^4^ in 24 well plates and co-transfected with a selected pre-miRNA and either pmirGLO-TTR-3′UTR -C or pmirGLO-TTR-3′UTR-G (500 ng) with the use of siPORT™ NeoFX™ Transfection Agent (Ambion) according to the manufacturer’s protocol. Cells were co-transfected with either of the pre-miR miRNA Precursor Molecules miR-622, miR-643, miR-337-3p and miR-325 (Applied Biosystems) in the concentrations 1 pmol, 10 pmol or 50 pmol. After 26 h, firefly and renilla activities were measured using a dual luciferase reporter assay (Promega) according to the manufacturer’s protocol on a FB12 Luminometer (Berthold detection system). Luciferase levels were compared to the levels in un-transfected cells to ensure proper transfection of the cells. The relative activity was calculated by normalization with the firefly activity. All experiments were made in triplicate.

To measure endogenous miRNA effects HepG2 cells were transfected as above, but without the miRNAs, and firefly and renilla activities were measured after 40 h.

### Bioinformatics and Statistics

The four tested miRNAs were identified previously [13]. In short, four different miRNA target prediction programs were used (MicroInspector 1.5, the PITA algorithm, RNAhybrid and RegRNA). Selection criteria was that all miRNA binding to the SNP should be identified using all four programs and at the same time not bind to the wild type allele.

All data was analyzed using the SPPS software (version 19). Independent samples t-test was used for assessing the differences between groups and a significant p-value was set to<0.05.

### Ethics Statement

The study was approved by the Regional Ethical Committee in Umeå (Dnr: 06–084 M). Informed written consent has been obtained from patients and clinical investigations were conducted according to the Declaration of Helsinki.

## Results

### mRNA Levels of Patients Carrying the SNP rs62093482-T Showed Similar Transcriptional Expression Pattern between the Mutated and Wild Type Allele

mRNA transcriptional expressions from the mutated and wild type allele were measured on nine liver biopsies from heterozygous Swedish V30M carriers. One of the samples was found to be homozygous for the rs62093482-T SNP but heterozygous for the V30M mutation and therefore used as a negative control. The allele specific expression of the mutated and wild type allele in the negative control was identical to each other as expected. However, in the heterozygous samples no difference in allele specific expression between the allele containing the V30M mutation and the rs62093482-T compared to the wild type allele was observed either (Figure 1a). When sorting the samples according to age at onset of disease, no difference between the early (<40 year) and the late (>50 year) onset cases was noted (data not shown).

### No Effect on Allele Specific Expression was seen in Transfection Experiments with miRNA

The pmirGLO-TTR-3′UTR -C, or the pmirGLO-TTR-3′UTR -T constructs were co-transfected into COS-7 cells, together with one of four miRNAs, respectively, in different concentrations and luciferase activity was measured. When introducing either the TTR-3′UTR-C or the TTR-3′UTR-T into the plasmid, luciferase activity went down probably due to the properties of the 3′ UTR. When co-transfected into COS-7 cells with the different miRNA no difference was found between the constructs, with or without miRNAs, independent of their concentration (Figure 2). This indicates that none of the tested miRNAs bind very well to the SNP in the 3′ UTR, and that they do not discriminate between the T-SNP and the wild type, and thus, have no effect on the translational or transcriptional regulation of TTR.

### Endogenous miRNA from a Liver Cell Line had No Effect on Allele Specific Expression of TTR

The pmirGLO-TTR-3′UTR -C, and the pmirGLO-TTR-3′UTR -T constructs, respectively, were transfected into a HepG2-cell line. As a control, a COS-7 cell line was also transfected. Luciferase activity was measured 40 hours after transfection. No difference in allele specific expression was detected neither in the COS-7 cell line (data not shown) nor in the HepG2 cell line (Figure 3).

## Discussion

In the last few years common polymorphisms in disease-causing genes has been shown to alter the phenotype and risk of developing disease. In the present study we, therefore, investigated if a SNP (rs62093482-C>T) found in the 3′ UTR in Swedish ATTR patients could act as a possible binding site for miRNA causing a down-regulation of variant TTR synthesis. Thereby this could contribute to the lower penetrance and later onset of disease observed in Swedish ATTR V30M population compared to other populations. This was the only SNP found in the 3′ UTR of the *TTR* gene and therefore of special interest. Two previous studies have reported that lower serum levels of mutated compared to wild type TTR, approximately 40% to 60%, are found in the Swedish ATTR V30M patients. [15], [16] In a similar Japanese study performed on Japanese V30M patients, the results were approximately 50% to 50% [17], indicating a difference between the two populations that should be elucidated. Although no serum TTR level concentrations were measured on our patients, we assume that their levels correspond to those previously reported for Swedish ATTR patients. However, our results disclosed no difference in mRNA levels between the disease and the wild type allele when measured in liver biopsies from Swedish ATTR patients. Even so, there still could be a regulation on the translational level since some miRNAs exert their function by binding to the mRNA and preventing translation rather than degrading the mRNA itself. Our luciferase assay measures light emitted from the firefly luciferase protein, that is transcribed from the firefly luciferase gene linked to either the TTR-3′UTR-C or TTR-3′UTR-T. Any differences in luciferase activity are thereby a result either of degradation of the transcript, or a translational regulation. Therefore we should have detected differences in expression in our study, but no differences between the alleles were found.

A finale experiment in the HepG2 cells indicates that no additional endogenous miRNA could affect the expression.

The differences in TTR protein levels in blood between mutated and wild type protein in Swedish ATTR patients are, therefore, not caused by a down-regulation on the transcriptional or translational level, but rather by decreased secretion of the variant protein from the endoplasmic reticulum (ER) and/or increased clearance from the circulation. In a previous study it was shown that the secretion of the V30M tetramer from the ER seemed to be equally efficient as the secretion of the non-mutated tetramer, indicating that impaired secretion of the mutated protein is not the cause for the difference in serum levels between mutated and normal TTR. [18] It is more likely that it is due to higher clearance of mutated TTR from the blood, which is supported by Benson et. al. [19] who demonstrated experimentally that V30M TTR is metabolized and excreted faster than wild type TTR. In a recent study of a Portuguese V30M population, the ratio of mutated to wild type TTR in plasma was approximately 40% to 60%, i.e. similar to that noted in the Swedish population. [20] This contributes to the conclusion that the SNP found in the 3′ UTR of TTR in Swedish V30M patients is non-functional, although the question still remains as to why there is a difference in serum levels between mutated and wild type TTR when the allelic expression is the same between the two alleles. Despite the knowledge that there are lower levels of mutated TTR circulating in the blood, our study is the first to investigate the allele specific expression of *TTR* in Swedish patients.

### Conclusions

We have shown that the SNP rs62093482-T has no effect in regulating the expression with the help of miRNA, endogenous or not, and thus can be ruled out as the cause of the lower penetrance seen in the Swedish ATTR population compared to other ATTR populations. However, this SNP found on the mutated *TTR* allele is unique for Swedish ATTR patients. It could therefore be utilized in identifying ATTR patients from other parts of the world originating from the Swedish founder mutation.

## References

[1] Kelly JW, Colon W, Lai Z, Lashuel HA, McCulloch J, et al (1997) Transthyretin quaternary and tertiary structural changes facilitate misassembly into amyloid. Adv Protein Chem 50: 161–181. 9338081 10.1016/s0065-3233(08)60321-6

[2] Plante-Bordeneuve V, Said G (2011) Familial amyloid polyneuropathy. Lancet Neurol 10: 1086–1097. 22094129 10.1016/S1474-4422(11)70246-0

[3] Plante-Bordeneuve V, Carayol J, Ferreira A, Adams D, Clerget-Darpoux F, et al (2003) Genetic study of transthyretin amyloid neuropathies: carrier risks among French and Portuguese families. J Med Genet 40: e120. 14627687 10.1136/jmg.40.11.e120 PMC1735318

[4] Hellman U, Alarcon F, Lundgren HE, Suhr OB, Bonaiti-Pellie C, et al (2008) Heterogeneity of penetrance in familial amyloid polyneuropathy, ATTR Val30Met, in the Swedish population. Amyloid 15: 181–186. 18925456 10.1080/13506120802193720 PMC2738945

[5] Coelho T ML, Martins A, Waddington Cruz M, Planté-Bordeneuve V, Suhr OB, et al. (2011) The long-term effects of tafamidis for the treatment of transthyretin type familial amyloid polyneuropathy. VIIIth International Symposium on Familial Amyloidotic Polyneuropathy.

[6] Coelho T SO, Adams D, Lozeron P, Hawkins P, Mant T, et al. (2011) Phase I safety, pharmacokinetic and pharmacodynamic results for ALN-TTR01, a novel RNAi therapeutic for the treatment of transthyretin amyloidosis. VIIIth International Symposium on Familial Amyloidotic Polyneuropathy. Kumamoto, Japan.

[7] Monia BP AE, Guo S, Benson MD (2011) Clinical development of an antisense therapy for the treatment of transthyretin-associated polyneuropathy. VIIIth International Symposium on Familial Amyloidotic Polyneuropathy. Kumamoto, Japan. 10.3109/13506129.2012.673140 22494066

[8] Kwak PB, Iwasaki S, Tomari Y (2010) The microRNA pathway and cancer. Cancer Sci 101: 2309–2315. 20726859 10.1111/j.1349-7006.2010.01683.x PMC11159795

[9] Liu Z, Wei S, Ma H, Zhao M, Myers JN, et al (2011) A functional variant at the miR-184 binding site in TNFAIP2 and risk of squamous cell carcinoma of the head and neck. Carcinogenesis 32: 1668–1674. 21934093 10.1093/carcin/bgr209 PMC3204352

[10] Amin AS, Giudicessi JR, Tijsen AJ, Spanjaart AM, Reckman YJ, et al. (2011) Variants in the 3′ untranslated region of the KCNQ1-encoded Kv7.1 potassium channel modify disease severity in patients with type 1 long QT syndrome in an allele-specific manner. Eur Heart J. 10.1093/eurheartj/ehr473 PMC3303714 22199116

[11] Wang G, van der Walt JM, Mayhew G, Li YJ, Zuchner S, et al (2008) Variation in the miRNA-433 binding site of FGF20 confers risk for Parkinson disease by overexpression of alpha-synuclein. Am J Hum Genet 82: 283–289. 18252210 10.1016/j.ajhg.2007.09.021 PMC2427225

[12] Laguette MJ, Abrahams Y, Prince S, Collins M (2011) Sequence variants within the 3′-UTR of the COL5A1 gene alters mRNA stability: implications for musculoskeletal soft tissue injuries. Matrix Biol 30: 338–345. 21609763 10.1016/j.matbio.2011.05.001

[13] Olsson M, Norgren N, Obayashi K, Plante-Bordeneuve V, Suhr OB, et al (2010) A possible role for miRNA silencing in disease phenotype variation in Swedish transthyretin V30M carriers. BMC Med Genet 11: 130. 20840742 10.1186/1471-2350-11-130 PMC2945965

[14] Soares ML, Coelho T, Sousa A, Holmgren G, Saraiva MJ, et al (2004) Haplotypes and DNA sequence variation within and surrounding the transthyretin gene: genotype-phenotype correlations in familial amyloid polyneuropathy (V30M) in Portugal and Sweden. Eur J Hum Genet 12: 225–237. 14673473 10.1038/sj.ejhg.5201095

[15] Holmgren G, Lundgren E, Kangawa K, Kurihara T, Matsukura S, et al (1993) Diagnostic radioimmunoassay and DNA-analysis in Swedish and Japanese patients with familial amyloidotic polyneuropathy. Homozygosity for the TTR met30 gene. Acta Neurol Scand 87: 124–127. 8095120 10.1111/j.1600-0404.1993.tb04090.x

[16] Suhr OB, Ando Y, Ohlsson PI, Olofsson A, Andersson K, et al (1998) Investigation into thiol conjugation of transthyretin in hereditary transthyretin amyloidosis. Eur J Clin Invest 28: 687–692. 9767365 10.1046/j.1365-2362.1998.00345.x

[17] Ueda M, Misumi Y, Mizuguchi M, Nakamura M, Yamashita T, et al (2009) SELDI-TOF mass spectrometry evaluation of variant transthyretins for diagnosis and pathogenesis of familial amyloidotic polyneuropathy. Clin Chem 55: 1223–1227. 19372189 10.1373/clinchem.2008.118505

[18] Sato T, Susuki S, Suico MA, Miyata M, Ando Y, et al (2007) Endoplasmic reticulum quality control regulates the fate of transthyretin variants in the cell. EMBO J 26: 2501–2512. 17431395 10.1038/sj.emboj.7601685 PMC1868898

[19] Hanes D, Zech LA, Murrell J, Benson MD (1996) Metabolism of normal and Met30 transthyretin. Adv Food Nutr Res 40: 149–155. 8858811 10.1016/s1043-4526(08)60025-x

[20] Ribeiro-Silva C, Gilberto S, Gomes RA, Mateus E, Monteiro E, et al (2011) The relative amounts of plasma transthyretin forms in familial transthyretin amyloidosis: a quantitative analysis by Fourier transform ion-cyclotron resonance mass spectrometry. Amyloid 18: 191–199. 22080762 10.3109/13506129.2011.614295

